# *Streptococcus pyogenes* antigen I/II-family polypeptide AspA shows differential ligand-binding properties and mediates biofilm formation

**DOI:** 10.1111/j.1365-2958.2011.07749.x

**Published:** 2011-07-15

**Authors:** Sarah E Maddocks, Christopher J Wright, Angela H Nobbs, Jane L Brittan, Linda Franklin, Nicklas Strömberg, Aras Kadioglu, Mark A Jepson, Howard F Jenkinson

**Affiliations:** 1 School of Oral and Dental Sciences, University of BristolBristol BS1 2LY, UK.; 2 Department of Cariology, Umeå UniversitySE-901 87 Umeå, Sweden.; 3 Department of Infection, Immunity and Inflammation, University of LeicesterLeicester LE1 9HN, UK.; 4 Wolfson Bioimaging Facility, and School of Biochemistry, University of BristolBristol BS8 1TD, UK.

## Abstract

The streptococcal antigen I/II (AgI/II)-family polypeptides are cell wall-anchored adhesins expressed by most indigenous oral streptococci. Proteins sharing 30–40% overall amino acid sequence similarities with AgI/II-family proteins are also expressed by *Streptococcus pyogenes*. The *S. pyogenes* M28_Spy1325 polypeptide (designated AspA) displays an AgI/II primary structure, with alanine-rich (A) and proline-rich (P) repeats flanking a V region that is projected distal from the cell. In this study it is shown that AspA from serotype M28 *S. pyogenes,* when expressed on surrogate host *Lactococcus lactis*, confers binding to immobilized salivary agglutinin gp-340. This binding was blocked by antibodies to the AspA-VP region. In contrast, the N-terminal region of AspA was deficient in binding fluid-phase gp-340, and *L. lactis* cells expressing AspA were not agglutinated by gp-340. Deletion of the *aspA* gene from two different M28 strains of *S. pyogenes* abrogated their abilities to form biofilms on saliva-coated surfaces. In each mutant strain, biofilm formation was restored by *trans* complementation of the *aspA* deletion. In addition, expression of AspA protein on the surface of *L. lactis* conferred biofilm-forming ability. Taken collectively, the results provide evidence that AspA is a biofilm-associated adhesin that may function in host colonization by *S. pyogenes*.

## Introduction

*Streptococcus pyogenes* (group A *Streptococcus*; GAS) is a causative agent of tonsillitis, pharyngitis, otitis media, impetigo, scarlet fever, cellulitis and necrotizing fasciitis. The species is part of a phenotypically heterogeneous group of bacteria that readily colonize humans and animals, ordinarily forming part of the commensal microbiota (Nobbs *et al*., 2009). Under appropriate conditions, *S. pyogenes* can cause opportunistic invasive infections with high (10–35%) mortality rates (NCIRD, 2008). In order to colonize, proliferate and persist, *S. pyogenes* must in the first instance adhere to host tissues. Numerous cell wall-anchored proteins have been identified on the surface of *S. pyogenes* (Nobbs *et al*., 2009) that mediate adherence to host tissue proteins such as fibronectin (Cue *et al*., 2001), fibrinogen (Hryniewicz *et al*., 1972; Courtney *et al*., 2002), albumin (Schmidt *et al*., 1993), immunoglobulins (Fagan *et al*., 2001; Okamoto *et al*., 2008) and mucin (Ryan *et al*., 2001). However, the mechanisms by which *S. pyogenes* colonizes oral or nasopharyngeal surfaces are not fully understood.

*Streptococcus pyogenes* has the potential to form biofilms in the oral cavity and nasopharynx (Doern *et al*., 2009), and on skin and wounds. Cases of pharyngitis from which biofilm-forming *S. pyogenes* have been isolated have been associated with multiple episodes of disease and 30% treatment failure (Lembke *et al*., 2006). It is clear that the ability of *S. pyogenes* to form biofilms varies considerably with respect to serotype and strain (Lembke *et al*., 2006; Courtney *et al*., 2009). One of the mechanisms proposed for initial adherence and accumulation of M1 serotype *S. pyogenes* involves the formation of complexes between cell surface lipoteichoic acid (LTA) and members of the M protein family (Courtney *et al*., 2009). Another more recent finding is that pilus structures (Mora *et al*., 2005) are involved in development of biofilm aggregates (Manetti *et al*., 2007; Koller *et al*., 2009). Clearly GAS utilizes different complements of surface proteins to adhere to host tissues and form biofilms.

In oral viridans group streptococci, the antigen I/II (AgI/II) family of cell surface proteins plays many roles in colonization of the host. The AgI/II-family polypeptides are cell wall-anchored and comprise between 1310 and 1653 amino acid (aa) residues (Brady *et al*., 2010). The SpaP (AgI/II-family) protein in *Streptococcus mutans* mediates adherence to salivary glycoproteins within the tooth enamel pellicle (Bowen *et al*., 1991). Antibodies to SpaP block adherence of *S. mutans* (Munro *et al*., 1993), and a peptide vaccine derived from SpaP has been shown to protect rodents against dental decay caused by *S. mutans* (Takahashi *et al*., 1991). Other indigenous streptococci, for example *Streptococcus gordonii*, express two AgI/II proteins, designated SspA and SspB, from duplicated genes (Jakubovics *et al*., 2005). The SpaP, SspA and SspB proteins have a common host receptor in gp-340, a member of the scavenger receptor cysteine-rich (SRCR) protein family (Madsen *et al*., 2010). Gp-340 is a mucin-like protein found in saliva, tears, and at mucosal surfaces. It functions in innate immune defence, complexing with other mucosal components such as mucins, collectins (Ligtenberg *et al*., 2007) and secretory immunoglobulin A (S-IgA) (Ligtenberg *et al*., 2004), and trapping microbes for removal from the body (Ligtenberg *et al*., 2007). However, when gp-340 becomes adsorbed to a surface, such as within salivary pellicle, gp-340 acts as a receptor for microbial adherence (Loimaranta *et al*., 2005). Additionally, AgI/II-family proteins have been shown to bind fibronectin (Nobbs *et al*., 2007), collagen (Soell *et al*., 2010), the oral bacteria *Actinomyces naeslundii* (Jakubovics *et al*., 2005) and *Porphyromonas gingivalis* (Daep *et al*., 2008), and the yeast *Candida albicans* (Silverman *et al*., 2010). These proteins therefore play potentially multiple roles in *Streptococcus* adherence, colonization and microbial community development.

**Fig. 1:**
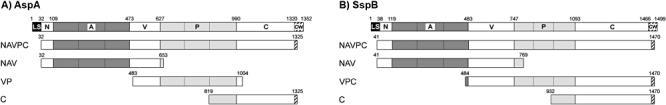
Pictorial representation of AgI/II proteins of (A) *S. pyogenes* serotype M28 (strain MGAS6180) and (B) *S. gordonii* DL1, showing the recombinant fragments generated in this study. Included are the aa residue numbers demarcating each of the protein fragments. Structural features are as follows: LS, leader sequence; N, N-terminal domain; A, alanine-rich repeats; V, variable region; P, proline-rich repeats; C, C-terminal domain; and CW, cell wall anchor.

Epidemiological studies of GAS associated with puerperal sepsis, a major cause of death of young women in the past, have identified serotype M28 strains as being largely responsible. The genome sequences of a series of M28 invasive strains revealed that they had all acquired a 37.4 kb element, shared with group B *Streptococcus* (GBS), designated region of difference 2 (RD2). This region contained genes encoding prophage virulence factors, R-28 surface protein antigen (Johnson, 1975; Stålhammar-Carlemalm *et al*., 1999) and six other inferred secreted proteins (Green *et al*., 2005). One of these genes encoded a protein (designated M28_Spy1325) with structural and sequence similarities to oral streptococcal AgI/II-family proteins (Fig. 1) (Jenkinson and Demuth, 1997). Preliminary characterization of the *S. pyogenes* AgI/II protein M28_Spy1325 (predicted molecular mass 148.36 kDa) indicated that it interacted with gp-340 in a manner consistent with that of other AgI/II-family proteins (Zhang *et al*., 2006). In addition, bioinformatic analyses revealed common ancestral characteristics of M28_Spy1325 (denoted AspA for group A*Streptococcus*surface protein A) that placed it firmly within the AgI/II-family of streptococcal proteins. Loss of sections of the N-terminal A and V regions has resulted in AspA being slightly smaller than many of the other oral streptococcal AgI/II proteins (Fig. S1) (Brady *et al*., 2010).

Although AspA binds gp-340 (Zhang *et al*., 2006), the primary structural differences between AspA and SspB suggested a differential role for AspA may have evolved in pathogenic GAS. To test this hypothesis we investigated the structural and functional properties of AspA in more detail, particularly in respect to binding gp-340 and in biofilm community development. We show that AspA expressed on the surrogate host *Lactococcus lactis* binds immobilized gp-340, but the N-terminal region is defective in interaction with fluid-phase gp-340. In addition, expression of AspA appears to be essential for biofilm formation by two independently isolated M28 serotype strains of *S. pyogenes*. It is suggested that expression of AspA may confer on GAS the ability to successfully compete with indigenous commensal streptococci in colonizing host surfaces while avoiding, at least in part, host innate immune pressures.

## Results

### Primary sequence differences between AspA and SspB

The AgI/II-family polypeptides have a common NAVPC region structure (Fig. 1), as described above, with highest conservation of aa sequences within the C-terminal region (Jenkinson and Demuth, 1997). The Ala-rich repeat (A) and Pro-rich repeat (P) regions also show various degrees of sequence conservation across the family, related to maintaining the N-terminal α-helix and C-terminal polyproline helix structures, which together generate a stalk projecting the V region distal from the cell surface-anchorage point (Brady *et al*., 2010; Larson *et al*., 2010). The GAS AgI/II polypeptide A and P regions show approximately 28% and 30% aa sequence identities, respectively, to the A and P regions of SspB from *S. gordonii* (see Fig. S1 for aa sequence alignments). The C regions of AspA and SspB have 38% aa residue identity, while the V region sequences appear to be unrelated (< 10% identical aa residues) (Fig. S1).

### Antigenic differences between AspA and SspB

Previous studies have investigated the binding properties of regions of AgI/II-family proteins by expressing recombinant fragments in *Escherichia coli* (Crowley *et al*., 1993; Hajishengallis *et al*., 1994). These studies have been paramount in deducing the 3D structure of AgI/II proteins (Forsgren *et al*., 2009; 2010; Brady *et al*., 2010; Larson *et al*., 2010). We have previously produced recombinant fragments of *S. gordonii* SspB and shown that these purified fragments have various binding activities with gp-340 (Nobbs *et al*., 2007). To compare therefore the binding properties of AspA regions with corresponding regions of SspB, we generated two sets of recombinant polypeptides (Fig. 1). The AspA polypeptides generated (Fig. 1A) were, as far as possible, analogous to those that had already been derived from SspB (Fig. 1B), except that we were unable to express a stable VPC fragment from AspA (see Fig. 1A).

**Fig. 2:**
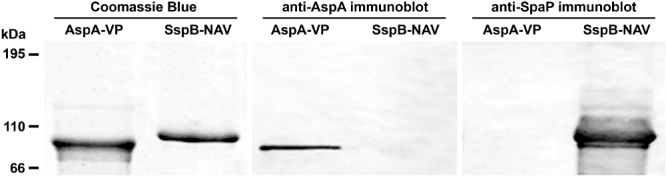
Western blots of recombinant protein fragments VP-AspA and NAV-SspB probed using anti-VP-AspA or anti-SpaP antibodies. Equivalent amounts of protein (100 ng) as determined by Bradford assay were applied to each lane. Proteins were also stained with Coomassie blue as a protein loading control.

Since the aa sequence of the V region of AspA was unique, we utilized fragment VP-AspA (Fig. 1A) to generate AspA-specific polyclonal antibodies. The reactivity of these antibodies with AspA or SspB protein fragments was compared with the reactivity of a polyclonal antibody raised to SpaP from *S. mutans* (Jakubovics *et al*., 2005). Anti-AspA-VP antibodies reacted as anticipated with the homologous antigen, recombinant VP (rVP-AspA) polypeptide (Fig. 2), but not with rNAV-SspB polypeptide (Fig. 2) or with rNAVPC-SspB (not shown). The anti-SpaP antibodies reacted with rNAV-SspB (Fig. 2) and with all of the recombinant SspB polypeptides i.e. rNAVPC, rVPC and rC (not shown). These observations were consistent with the relatively well-conserved sequences across the entire lengths of the SpaP and SspB proteins, with the exception of the V regions (Brady *et al*., 2010). Conversely, the SpaP antibodies did not react with any of the AspA recombinant polypeptides, reflecting the lower aa sequence identities between AspA and SspB, and suggesting major antigenic distinctions between the two polypeptides.

### Metal ion binding associated with V region

**Fig. 3:**
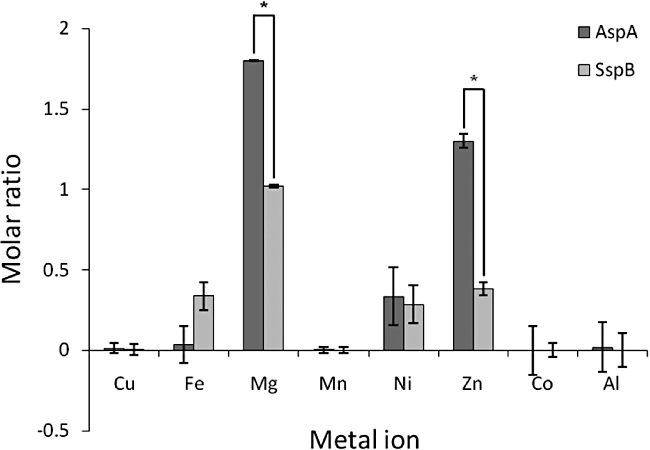
Metal ion analysis of recombinant protein fragments NAV-AspA (dark grey bars) and NAV-SspB (light grey bars) by ICP-OES using ICP multi-element standard solution IV (Merck) and 100 ng µl^−1^ protein. Values are metal ion : protein ratios comparative to a buffer standard containing no protein. Error bars represent ± SD from three independent experiments. *P < 0.001 between samples as indicated.

Within the V region aa sequence of AspA there is a His/Asp motif, HDDWVHDTH (608–616 aa), which is not annotated in any protein databases, and that is found only in the AgI/II-family proteins identified in GAS and GBS. It is known that clustered His residues form a region of high electron density to which positively charged metal ions may bind. Accordingly, inductively coupled plasma optical emission spectrometry (ICP-OES) was utilized to determine trace metal ion binding to the rNAV polypeptides. The analyses found that Zn^2+^ ions bound to rNAV-AspA at a metal ion : protein ratio of approximately 1:1 (Fig. 3). However, ICP-OES analysis of rNAV-SspB, which does not contain the HD cluster, suggested that this region did not specifically bind Zn^2+^ ions over and above similar amounts of residual Fe^2+^ and Ni^2+^ associated with the protein (Fig. 3). In addition, Mg^2+^ (or Ca^2+^) was found to be associated with both AspA and SspB polypeptides at metal ion : protein ratios of approximately 2:1 and 1:1 respectively (Fig. 3). Under the ICP-OES conditions used, it was not possible to distinguish between Mg^2+^ and Ca^2+^. Zinc (Zn^2+^) has not previously been identified as being associated with the AgI/II-family protein V regions. The residual amounts of Ni^2+^ present in both protein preparations (Fig. 3) may have arisen from the Ni^2+^ affinity column utilized to purify the His-tagged polypeptides.

### Interactions of fluid-phase gp-340 with AspA fragments

**Fig. 4:**
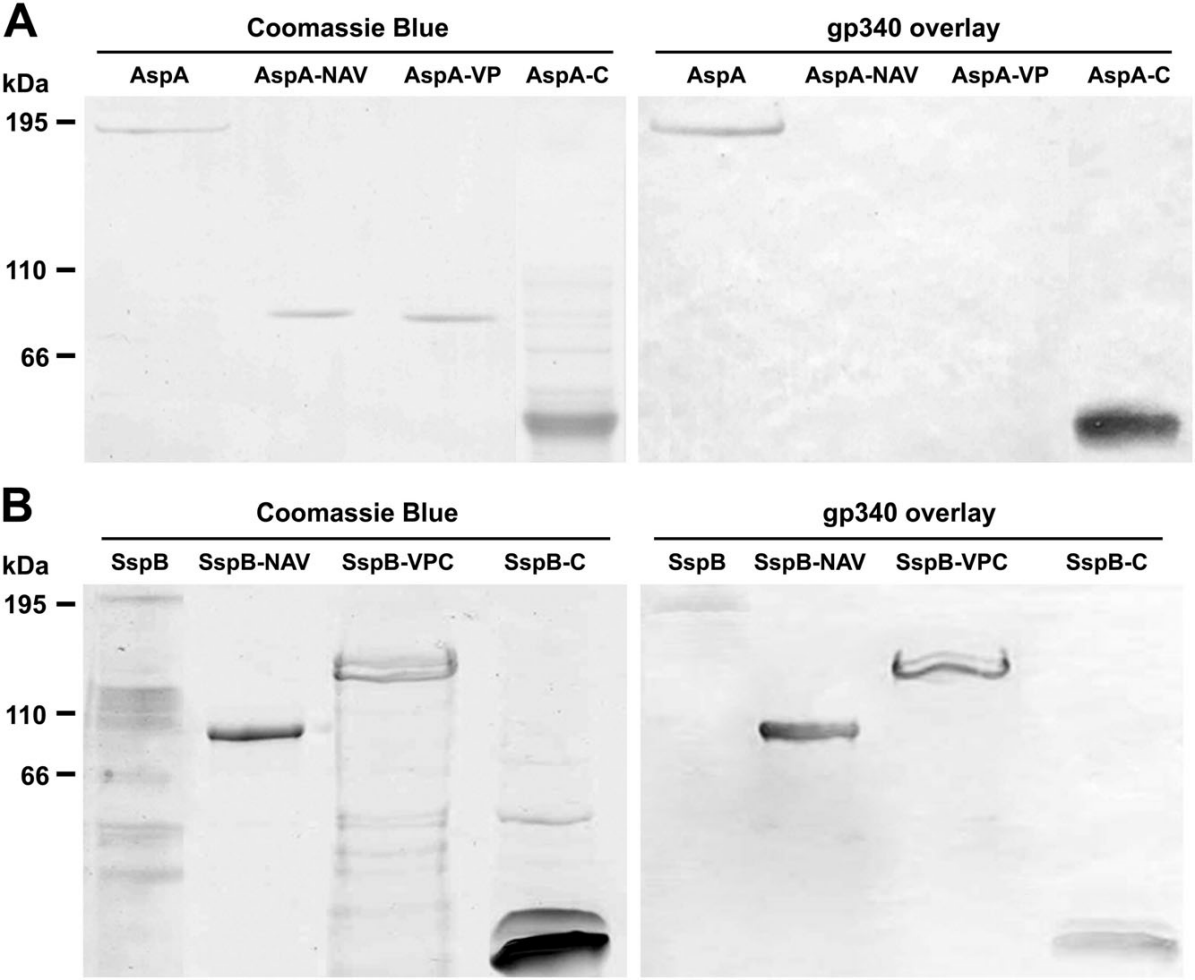
Binding of fluid-phase gp-340 by recombinant AspA or SspB protein fragments. SDS-PAGE patterns of proteins stained with Coomassie blue (left-side panels) and corresponding far-Western blots of (A) AspA-recombinant proteins AspA (NAVPC), NAV, VP and C, and (B) SspB-recombinant proteins SspB (NAVPC), NAV, VPC and C. Recombinant proteins (100–200 ng) were resolved by SDS-PAGE, blotted onto nitrocellulose and overlayered with 100 ng gp-340 ml^−1^. Binding of gp-340 to the bands was detected by probing with mouse monoclonal gp-340 antibody followed by HRP-conjugated goat anti-mouse antibody.

By utilizing the recombinant polypeptides described in Fig. 1, we then determined in far-Western blot overlays with gp-340 which of the various recombinant polypeptides were able to interact with gp-340. In these experiments, binding of gp-340 to the blotted polypeptides was determined using monoclonal antibody to gp-340 polypeptide backbone and anti-mouse HRP-conjugated secondary antibody. All of the SspB fragments, i.e. rNAV, rVPC and rC, and full-length rSspB, were found to bind gp-340 (Fig. 4). Purified full-length rSspB was subject to degradation and tended to bind gp-340 rather weakly. In contrast, only the full-length rAspA and rC-AspA polypeptides bound fluid-phase gp-340, while the rNAV-AspA and rVP-AspA polypeptides did not (Fig. 4). There were some apparent discrepancies between predicted and observed molecular masses on SDS-PAGE for some of these polypeptides. Anomalous migration on SDS-PAGE is common for polypeptides that are largely α-helical, e.g. A region, or that contain regions of random coils, e.g. P region. The C regions, on the other hand, resolved with predicted molecular mass of approximately 55 kDa (Fig. 4).

### Binding of *L. lactis* expressing AspA to immobilized gp-340

**Fig. 5:**
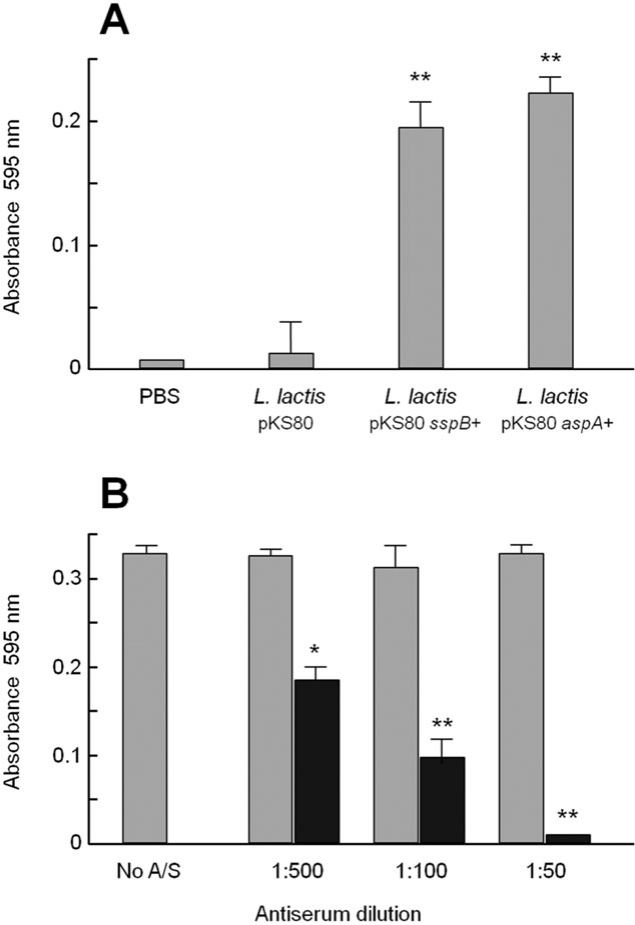
Interactions of *L. lactis* strains expressing AspA or SspB with immobilized gp-340 (50 ng). A. Binding of *L. lactis* MG1363 (pKS80) control cells, *L. lactis* (pKS80 *sspB*^+^) and *L. lactis* (pKS80 *aspA*^+^). Data represent the average ± SD of three independent experiments with triplicate samples. **P < 0.005 relative to *L. lactis* pKS80. B. Effect of dilutions of SpaP antiserum (grey fill) or rVP-AspA antiserum (black fill) on adherence of *L. lactis* (pKS80 *aspA*^+^) cells to gp-340. Control indicates no antiserum added. Input cell numbers in all experiments were 5 × 10^7^ cells well^−1^. Error bars are ± SD of two independent experiments with duplicate samples. **P < 0.005, *P < 0.05, relative to no antiserum control.

The structurally conserved A, P and C regions of AgI/II-family proteins are present in AspA and SspB, and sequences within these three regions in SspB have all been shown to interact with gp-340 (Brady *et al*., 2010). To determine the ability of AspA to bind immobilized gp-340, we cloned the *aspA* gene, and separately the *sspB* gene, into the expression vector pKS80 (Hartford *et al*., 2001) that replicates in *L. lactis*. The abilities of *L. lactis* MG1363 strains expressing AspA (pKS80 *aspA*^+^) or SspB (pKS80 *sspB*^+^) to adhere to gp-340 were compared with control *L. lactis* MG1363 containing empty vector, utilizing a crystal violet spectrophotometric assay. Binding levels of *L. lactis* expressing AspA to immobilized gp-340 were found to be similar to those of *L. lactis* expressing SspB (Fig. 5A), while the control cells showed < 5% binding of the protein-expressing strains.

Although only the C-terminal fragment of AspA bound fluid-phase gp-340 on far-Western blot overlays, it is known that fluid-phase gp-340 presents different receptor conformations compared with immobilized gp-340 (Loimaranta *et al*., 2005). Accordingly, to determine if the VP region of AspA carried sequences recognizing immobilized gp-340, we investigated the effects of rVP-AspA antibodies on adherence of *L. lactis* (pKS80 *aspA*^+^) to immobilized gp-340. It was found that antiserum raised to rVP-AspA inhibited adherence of *L. lactis* (pKS80 *aspA*^+^) cells to gp-340 in a dose-dependent manner (Fig. 5B), whereas corresponding dilutions of antiserum raised to *S. mutans* SpaP, which does not react with AspA (Fig. 2), had no inhibitory effects (Fig. 5B). Antiserum against rVP-AspA had no significant inhibitory effect on adherence of *L. lactis* (pKS80 *sspB*^+^) to immobilized gp-340 (data not shown).

### Aggregation properties of *L. lactis* cells expressing AspA

**Fig. 6:**
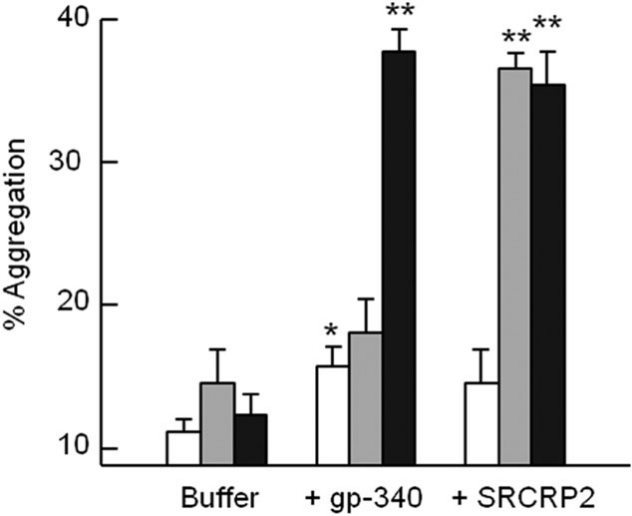
Aggregation of *L. lactis* strains expressing AspA or SspB in the presence of gp-340 or SRCRP2 peptide. Cells (10^8^ cells ml^−1^) of each strain were incubated with 100 ng ml^−1^ gp-340 or 100 ng ml^−1^ SRCRP2 peptide, or in PBS alone, at 37°C for up to 5 h. Optical density (600 nm) was measured at intervals over 5 h and % aggregation was calculated from change in OD_600_, corrected for OD_600_ decrease in controls. Columns are *L. lactis* MG1363 (open), *L. lactis* (pKS80 *aspA*+) (grey fill) and *L. lactis* (pKS80 *sspB*+) (black fill). The data represent the average ± SD of three independent experiments with triplicate samples. **P < 0.005, *P < 0.05, relative to respective buffer only controls.

To then test the ability of AspA to mediate aggregation of *L. lactis* by gp-340, aggregation of lactococcal suspensions mixed with gp-340 was determined. Assays were also performed with a peptide, designated SRCR Peptide 2 (SRCRP2), in place of gp-340. This peptide comprises a 16-aa-residue sequence found within the SRCR domains of the backbone of gp-340 (Leito *et al*., 2008). The peptide is reported to be active in aggregating a wide range of bacteria and it also interacts with AgI/II-family proteins (Jakubovics *et al*., 2005). In aggregation assays with gp-340, it was found that *L. lactis* cells expressing SspB were strongly aggregated in the presence of gp-340 while *L. lactis* cells expressing AspA were only weakly aggregated (Fig. 6). On the other hand, *L. lactis* cells expressing AspA or SspB were aggregated equally well in the presence of SRCRP2 (Fig. 6). These results indicate that the overall mechanism involved in cell aggregation as mediated by SRCRP2 is different from the mechanism associated with gp-340-mediated cell aggregation. Moreover, these gp-340-mediated aggregation data are in keeping with the above experimental results showing that the N-terminal region of AspA did not bind fluid-phase gp-340 (Fig. 4). Although the C regions of AspA and SspB were both shown to bind fluid-phase gp-340 (Fig. 4), clearly this interaction alone is not sufficient to promote aggregation of *L. lactis* (pKS80 *aspA*^+^) cells by gp-340.

An additional property of SspB polypeptide is that it mediates co-aggregation between *S. gordonii* and *A. naeslundii* (Jakubovics *et al*., 2005). When expressed on the surface of *L. lactis*, SspB confers the ability on *L. lactis* to co-aggregate with *A. naeslundii* T14V (renamed *A. oris* T14V). In a similar manner, SspA from *S. gordonii* expressed on *L. lactis* confers co-aggregation with *A. naeslundii* PK606 but not with strain T14V (Jakubovics *et al*., 2005). *L. lactis* expressing AspA showed no co-aggregation ability with six different *Actinomyces* strains, including T14V and PK606 (data not shown). It is concluded that AspA does not interact with *A. naeslundii*, a major bacterial component of early dental plaque and of other oral biofilm communities (Palmer *et al*., 2003).

### Role of AspA in GAS biofilm formation

Since various AgI/II-family adhesins have been implicated in mediating adherence and biofilm formation (Pecharki *et al*., 2005; Ahn *et al*., 2008), we investigated the abilities of two serotype M28 strains of *S. pyogenes*, MGAS6180 and H360, to form biofilms on salivary pellicle. Knockout mutants of these strains were generated as described in *Experimental procedures* and were then complemented *in trans* (see Fig. S2A). The allelic replacement resulted in deletion of the complete coding sequence, leaving in place the native promoter and terminator, as confirmed by sequencing. Moreover, sequencing determined that the deduced aa sequence of AspA from strain H360 was identical to that from MGAS6180.

To confirm that the *aspA* gene was inactivated, cell wall proteins were extracted from strains MGAS6180 and H360 following incubation with mutanolysin/lysozyme, separated by SDS-PAGE, and Western blots of proteins were incubated with rVP-AspA antibodies. The AspA protein in wild-type strain extracts appeared as a single immunoreactive band (Fig. S2B), and this band was absent from extracts of strains MGAS6180 Δ*aspA* and H360 Δ*aspA*. Cell wall extracts of complemented strains MGAS6180 Δ*aspA* (pKS80 *aspA*^+^) and H360 Δ*aspA* (pKS80 *aspA*^+^) both showed presence of many-fold higher levels of AspA protein than respective wild-type strains (Fig. S2B).

The Δ*aspA* mutant strains of MGAS6180 and H360 were identical in colony morphology to the respective parent strains, and had similar growth rates in minimal C medium to the parent strains. In adherence assays to immobilized gp-340, the mutants did not show any significant differences in adherence levels compared with respective wild-type (results not shown). This phenotype was not unexpected since at least two other proteins on the surface of GAS are known to interact with gp-340 (Edwards *et al*., 2008; Loimaranta *et al*., 2009).

**Fig. 7:**
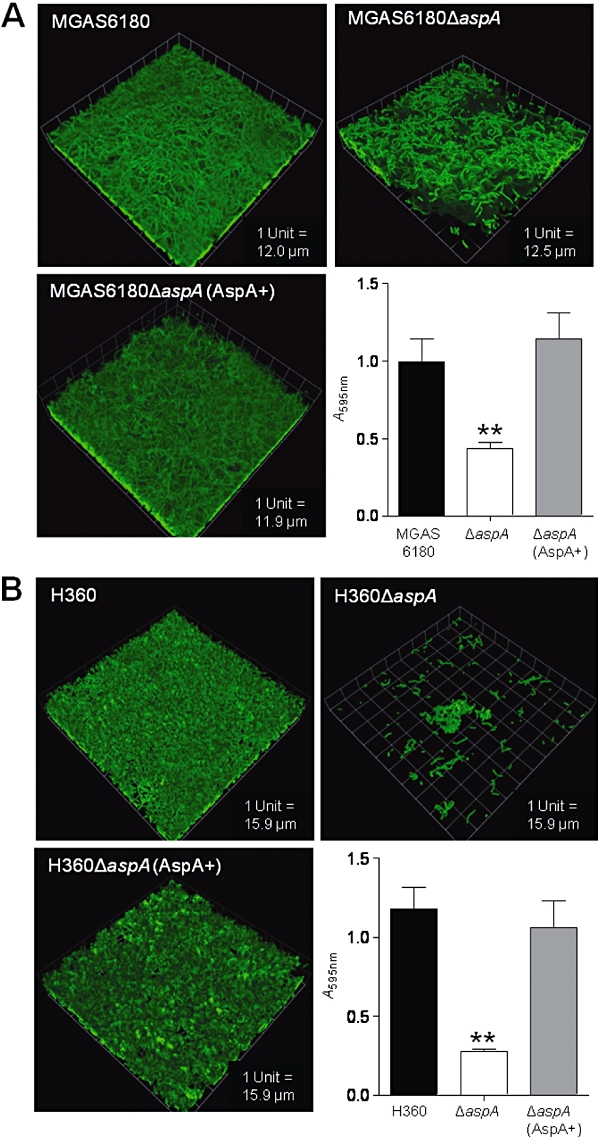
Confocal laser scanning microscopy images and biomass measurements of 24 h biofilms formed on salivary pellicle-coated coverslips, comparing biofilm structures of (A) *S. pyogenes* MGAS6180 and (B) strain H360, and corresponding isogenic Δ*aspA* mutants and complemented strains Δ*aspA* (*aspA*^+^). Biomass data were obtained by crystal violet assay (*A*_595_) of corresponding biofilms formed on coverslips. One unit is equivalent to the grid square side length. Data are averages ± SD of three independent experiments. **P < 0.005 relative to MGAS6180 (A) or H360 (B) wild-type strains.

Confocal laser scanning microscopy (CLSM) was employed to investigate differences in the development and structure of *S. pyogenes* biofilms as a consequence of deleting *aspA*. Wild-type strain MGAS6180 formed a biofilm after 24 h that was about 25 µm thick, consisting of densely packed cells with very little of the underlying salivary pellicle substratum visible (Fig. 7A). The corresponding knockout mutant, MGAS6180 Δ*aspA*, showed a much reduced biofilm thickness, with areas of the pellicle substratum clearly visible, and an approximately 60% reduction in biomass. Biofilms produced by strain H360 after 24 h were also approximately 25 µm thick with an ordered structure comprised of densely packed cells (Fig. 7B). The H360 Δ*aspA* knockout mutant was severely deficient in biofilm formation, with only small clumps of cells adhering to the pellicle substratum and a > 80% reduction in biomass (Fig. 7B). The complemented mutants were fully restored in biofilm formation, in each case reaching similar levels of biomass to the wild-type strains (Fig. 7). However, the architectures of the complemented strain biofilms were more disorganized than wild-type strain biofilms and the cells appeared to be more loosely packed (Fig. 7). These observations strongly suggest that AspA production is essential for biofilm formation by these two M28 serotype strains of GAS.

**Fig. 8:**
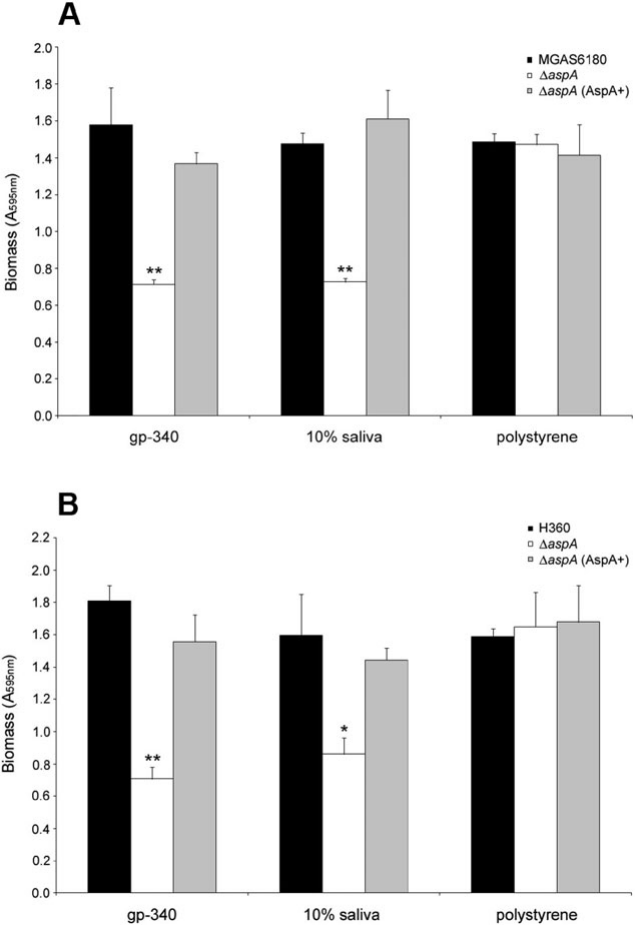
Biofilm formation on gp-340, saliva and polystyrene by (A) *S. pyogenes* MGAS6180 and (B) strain H360 (black fill), and corresponding isogenic Δ*aspA* mutants (no fill) and complemented strains Δ*aspA* (AspA+) (grey fill). Biomass data were obtained by crystal violet (*A*_595_) assay. Values given represent mean ± SD of three biological replicates from an experiment repeated twice. **P < 0.01, *P < 0.05, relative to respective MGAS6180 or H360 wild-type strains.

To further define the specificity of AspA in mediating biofilm formation, the abilities of Δ*aspA* and complemented mutants to form biofilms on gp-340, saliva or polystyrene compared with wild-type strains were investigated. Total biomass measurements for each wild-type strain on the three substrata were not significantly different (Fig. 8). However, Δ*aspA* mutants of strains MGAS6180 and H360 were significantly impaired in biofilm formation on gp-340 compared with wild-type, with 60% reduced biomass (Fig. 8A and B). Likewise, Δ*aspA* mutant biofilms formed on 10% saliva-coated polystyrene showed reduced biomass by approximately 50%. In contrast, there were no significant differences in biofilm production on polystyrene between wild-type strains and their respective Δ*aspA* mutants (Fig. 8A and B). The complemented strains exhibited similar levels of biofilm formation (biomass) to wild-type strains on all three substrata tested (Fig. 8). These results suggest AspA specificity for salivary glycoprotein substratum.

### AspA directly enhances biofilm development

**Fig. 9:**
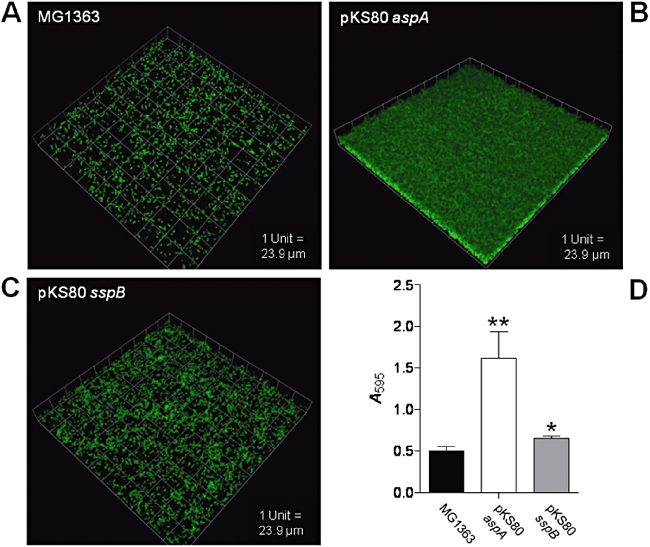
Confocal laser scanning microscopy images and corresponding biomass measurements of 24 h biofilms formed on salivary pellicle-coated coverslips of (A) wild-type *L. lactis* MG1363; (B) *L. lactis* (pKS80 *aspA*^+^); and (C) *L. lactis* (pKS80 *sspB*^+^). One unit is equivalent to the grid square side length. Biomass results (D) were obtained by crystal violet assay (*A*_595_) of corresponding biofilms formed on coverslips. The biomass data are averages ± SD of three independent experiments. **P < 0.005, *P < 0.05, relative to *L. lactis* MG1363 wild-type.

To confirm that AspA plays a direct function in promoting adherence and biofilm formation, the effects of expressing AspA in *L. lactis* on biofilm formation were investigated. Wild-type strain MG1363 cells produced a very sparse biofilm on salivary pellicle after 24 h incubation (Fig. 9A). However, *L. lactis* (pKS80 *asp*A^+^) expressing AspA protein produced a biofilm approximately 26 µm thick of densely packed cells (Fig. 9B), with more than threefold higher biomass (Fig. 9D). We also compared the ability of SspB protein to promote biofilm formation in *L. lactis*. Expression of SspB polypeptide led to a slight increase in coverage of the substratum by *L. lactis* (pKS80 *sspB*^+^) compared with wild-type *L. lactis* (Fig. 9C and D), but not to the same extent as expression of AspA did (Fig. 9B and D). This result may support the notion that AgI/II proteins may be less intimately involved in biofilm formation by *S. gordonii* (Zhang *et al*., 2005). Collectively, the results provide strong evidence that AspA is directly involved in GAS biofilm development upon salivary glycoprotein substrata. In addition, while AspA appears to differ functionally compared with some oral AgI/II-family polypeptides, it is suggested that AspA may constitute a novel colonization factor in GAS.

## Discussion

The AgI/II proteins have been a focus in studies of the abilities of oral viridans streptococci to colonize oral cavity and nasopharyngeal surfaces, and to cause tooth decay. A primary host factor shown to be recognized by the AgI/II-family proteins is the mucin-like glycoprotein designated salivary agglutinin (or gp-340). The significance of this interaction is believed to be at least double-edged: gp-340 acts in concert with other innate factors, such as mucins, collectins and defensins, to restrict the growth of commensal bacteria, while at the same time providing a potential substratum for adhesion of bacteria to oral cavity surfaces. Some oral streptococci, e.g. *S. mutans*, depend strongly upon AgI/II protein for adherence, evidenced by analyses of AgI/II gene knockout mutants that are rendered deficient in adherence to salivary glycoprotein pellicle (Koga *et al*., 1990). Other oral streptococci, such as *S. gordonii*, appear to have evolved a wider repertoire of salivary pellicle adhesins (Zhang *et al*., 2005), and so AgI/II-deficient mutants of these organisms may not be noticeably affected in adherence (Jenkinson *et al*., 1993). While the structural features and biological properties of these proteins are to various degrees conserved, their biological functions in respect of microbial physiology and ecology may be quite different depending upon the complement of adhesins that are expressed.

The overall structure of the AgI/II-family proteins may be envisaged in terms of a head (V) region (Troffer-Charlier *et al*., 2002), projected by a semi-flexible stalk (formed by interactions of the A and P regions) linked to a wall-proximal basal C-region (Brady *et al*., 2010). In the SspB protein there is evidence that the head (V) and stalk (A–P) regions provide gp-340-binding activity, and that the NAV region functions in SspB-mediated co-aggregation with *A. naeslundii* (Jakubovics *et al*., 2005). The C-terminal region of SspB also has gp-340-binding activity, and carries a sequence (designated BAR) that is recognized by the periodontal disease-associated bacterium *P. gingivalis* (Daep *et al*., 2008). The results in this article suggest that the AspA N-terminal region does not interact in the same way with fluid-phase gp-340, although the C-terminal region retains gp-340-binding sequences. These results are somewhat different from those previously published (Zhang *et al*., 2006), where it was shown that N-terminally derived fragments of M28_Spy1325 bound fluid-phase gp-340. One reason for this discrepancy could be related to the different gp-340 preparations being employed in the two studies. In our study, gp-340 was purified from parotid saliva, which contains no mucins, by adsorption to *S. mutans* cells expressing AgI/II adhesin. However, Zhang *et al*. (2006) utilized whole saliva (from several glands) and adsorption to *S. pyogenes* cells expressing a range of salivary glycoprotein adhesins. Secretory IgA is known to be present in some gp-340 preparations and could bind, complexed with gp-340, to AspA (Ligtenberg *et al*., 2004). However, in the case of our gp-340 preparations, S-IgA is not present at the level of chemical detection (Loimaranta *et al*., 2005). Regardless of the reasons for the discrepancy, the present data suggest a C-terminally located AspA binding site for fluid-phase gp-340, while Zhang *et al*. (2006) indicate there may be multiple gp-340 binding sites in AspA.

The observation that the N-terminal region of AspA does not apparently bind fluid-phase gp-340 would be in keeping with some of the features of the AspA primary sequence that differ from those of SspB. The N-terminal region of SpaP (and of SspB) has been shown to contain a series of linear aa sequences that interact with salivary glycoproteins (SGPs) (Brady *et al*., 2010). These sequences are depicted as SGP1 and SGP2 in Fig. S1 and comprise SGP1 (TYEAALKQYEADL), repeated four times with some conserved aa residue changes (Okahashi *et al*., 1993), and SGP2 (TELARVQKANADAKAAY), repeated three times with various conserved aa residue changes (Moisset *et al*., 1994). In AspA, these SGP-binding sequences are poorly conserved. The corresponding SGP1 sequences in AspA contain only between two and five identical aa residues respectively out of 13 within SspB (15–38%), while the SGP2 sequences in AspA contain between two and five identical aa residues out of 17 aa residues within SspB (12–29%). Although *L. lactis* cells expressing AspA were not enhanced in gp-340-mediated aggregation, they were agglutinated by SRCRP2 peptide (Bikker *et al*., 2002), confirming that the two aggregation reactions occur by different mechanisms.

On the other hand, the ADH1 and ADH2 sequences identified by Kelly *et al*. (1995) within the C-terminal region of SpaP that bind gp-340 are much better conserved in AspA (Fig. S1). These sequences in AspA and SspB have 47–50% identical aa residues. Potentially therefore the gp-340-binding regions within the C-terminus have been relatively well conserved in AspA, and this is supported by the far-Western blot data showing that the C-terminal region of AspA binds gp-340.

The expression of *aspA* within the surrogate host *L. lactis* showed clearly that the expressed protein mediated adherence to immobilized gp-340, in a similar manner to SspB. This appeared to be dependent upon the VP region, since rVP-specific antibodies blocked adherence to gp-340. Antibodies to the V region of SpaP have also been shown to block adherence of *S. mutans* to gp-340 (Brady *et al*., 1992). This finding indicates that the V region of *S. pyogenes* AspA might be a target when devising new means to inhibit colonization of mucosal surfaces by *S. pyogenes.*

The SspA and SspB proteins in *S. gordonii* interact with other bacteria and promote the formation of mixed species communities. The N-terminal domains have been shown to mediate co-aggregation of *S. gordonii* with *A. naeslundii* in the formation of early microbial communities, with the V regions conferring strain specificity (Jakubovics *et al*., 2005). We were unable to demonstrate that *L. lactis* cells expressing AspA interacted with *A. naeslundii*. This suggests that targeting of the V region of *S. pyogenes* AspA in preventive strategies might not affect development of oral biofilm communities.

The differential interactions of AspA and SspB with gp-340 could be related to the putative metal ion-binding sites within the V regions. The V region of SspB has recently been modelled with the structure resolved to 2.3 Å (Forsgren *et al*., 2009) to contain a Ca^2+^ molecule. However, Mg^2+^ was shown to also fit this model as an alternative metal ion. Since Ca^2+^ and Mg^2+^ have a similar charge and are of a similar size, and they were not distinguishable under the ICP-OES conditions used, they could be interchangeable at this position. A main finding was that AspA, but not SspB, bound one Zn^2+^ ion per protein molecule. Binding of Zn^2+^ ions is often associated with four aa residue ligands (His, Asp, Glu or Cys) (Vallee and Auld, 1993; Auld, 2001). A putative Zn^2+^ binding site is found at 608–616 aa residues in AspA, and comprises three His and three Asp residues (Fig. S1). In addition, a number of potential, alternative ligand (His) aa residues are located distally, between 20 and 129 aa residues away from the metal ion-binding site, as is typical for Zn^2+^ binding protein domains (Andreini *et al*., 2006). None of these potential Zn^2+^ binding residues is present within SspB or within *Enterococcus faecalis* aggregation substance protein (Hendrickx *et al*., 2009), to which the V region of AspA has 35–40% identity. Thus the N-terminal region of AspA appears to have diverged such that it contains at least one new metal ion (Zn^2+^) binding site.

The Zn^2+^ could have a structural or catalytic role. Histidine residues are similarly positioned within Zn^2+^ binding sequences in other streptococcal proteins such as pneumococcal zinc metalloproteinase (Iga) (Bender and Weiser, 2006) and metallopeptidase (Froeliger *et al*., 1999). A gp-340-binding protein, designated StcE, identified in enterohaemorrhagic *E. coli* has a histidine-rich Zn^2+^ binding site necessary for interaction and enzymatic degradation of gp-340 (Lathem *et al*., 2002; Grys *et al*., 2005). We have not been able to show that AspA has proteolytic activity towards gp-340, under a wide range of conditions. Nor has it been possible to demonstrate proteolytic activity of rAspA on elastin, gelatin, collagen or fibrinogen (data not shown). We are currently investigating further the properties and functions of the V region in AspA.

If bacteria in the oral cavity are rapidly aggregated by gp-340 they may be cleared from the host before becoming established within communities on the hard or soft tissue or prosthetic surfaces available. Microorganisms that are able to better evade innate defences such as agglutination and phagocytosis will be able to colonize and develop a niche that, in the case of GAS, may lead to chronic infection. It is suggested that AspA on the *S. pyogenes* cell surface binds to gp-340 present on oral cavity or nasopharyngeal surfaces, as does SspB expressed by *S. gordonii*, promoting colonization. The SspB protein is then able to bind fluid-phase gp-340, promoting biofilm formation through intermolecular bridging of adhesins. AspA appears to promote biofilm community formation, at least in part, through cell–cell interactions mediated directly by AspA. Notably, AspA polypeptide expression was necessary for biofilm formation on gp-340 or salivary glycoproteins adsorbed to plastic (polystyrene), but not for biofilm formation on the plastic itself. This suggests that the bacteria respond to the physiological substratum (salivary glycoproteins). The mechanism by which this might occur is currently not known, but *aspA* could be upregulated in response to salivary glycoproteins, which would be in keeping with evidence that the *S. gordonii sspA* and *sspB* genes are upregulated in response to saliva (Dû and Kolenbrander, 2000).

The reduced ability of AspA to bind fluid-phase gp-340 could suggest that GAS cells are less likely to be subject to innate defence recognition. Since scavenger receptor-like (SRCR) sequences are also expressed on the surfaces of macrophages and of other immune cells (Sarrias *et al*., 2004), it could be that reduced binding to various forms of these receptors may further enable GAS to more effectively evade immune defences. This possibility is currently under investigation. In conclusion it is suggested that AspA provides *S. pyogenes* with a new salivary pellicle or mucosal surface adhesin that increases GAS competitiveness with indigenous or commensal microbiota to generate a niche. Although the pilus structures in GAS serotype M1 strain S370 have been shown to be necessary for biofilm formation (Manetti *et al*., 2007), pili do not appear to be essential for biofilm development by the two M28 strains used in this work. Direct AspA protein–protein interactions are probably responsible for enhanced biofilm formation and GAS community development. This could provide increased protection *in vivo* against killing by antibiotics or advance pathogenicity by protecting against phagocytic cells. Taken collectively, these studies identify AspA as a potential new colonization factor in GAS serotype M28 and other *aspA*-carrying strains that could be a novel target for intervention.

## Experimental procedures

### Bacterial strains

Strains used in this study are outlined in Table S1. *E. coli* strains JM109 and XL1 were used for molecular cloning experiments to generate pET vector- or pGEM-T-based constructs, and *E. coli* BL21/λDE3 (Novagen) for expressing recombinant proteins. *E. coli* was cultured in LB broth at 37°C with shaking. *Streptococcus* strains were grown in Todd–Hewitt broth (Oxoid) containing 0.5% yeast extract. *Actinomyces* strains were grown in TY-glucose medium (Jakubovics *et al*., 2005). *S. pyogenes* strains were cultured at 37°C in an atmosphere of 5% CO_2_ while all other *Streptococcus* and *Actinomyces* strains were grown in a candle jar at 37°C. *L. lactis* strains were routinely cultured in M17 medium containing 0.5% glucose in a candle jar at 30°C. Bacto agar (Difco) was added at 1.4% final concentration as appropriate. For analysis of aggregation properties, *L. lactis* strains were grown in C medium containing 0.2% glucose (Lyon *et al*., 1998). Antibiotics were added to media at the following concentrations: ampicillin, 100 µg ml^−1^ and kanamycin, 50 µg ml^−1^ for *E. coli*; erythromycin, 10 µg ml^−1^ for *L. lactis*; spectinomycin, 100 µg ml^−1^ and erythromycin, 5 µg ml^−1^ for *S. pyogenes*.

### Recombinant protein production and purification

Chromosomal DNA was extracted from *S. pyogenes* MGAS6180 as described previously (Jenkinson, 1987). Primers (Sigma Aldrich) were designed to amplify regions of the locus *spy1325* (*aspA*) corresponding to the full-length AspA protein excluding the leader sequence and cell wall anchor motif (AspA-F1 & AspA-R1), NAV region (AspA-F1 & AspA-R3), VP region (AspA-mF & AspA-mR) and C region (AspA-F3 & AspA-R1) of AspA (Table S2; Fig. S1). Additional sequence was incorporated at the 5′ end of both the forward (gacgacgacaagatx) and reverse primers (gaggagaagcccggtxx), to enable the PCR products to be cloned into the ligation-independent expression vector pET46-Ek-LIC (Novagen). PCR utilized a proofreading enzyme (Bio-X-Act Long; Bioline) and the PCR products were treated with T4 polymerase (Novagen) prior to mixing with pET46, according to the manufacturer's instructions. Fragments cloned into pET46 incorporated a sequence encoding a 6x His-tag at the N-terminus of the polypeptide.

Expression vectors were transformed into chemically-induced competent *E. coli* BL21/λDE3 (Novagen), prepared by the method of Hanahan (1983). Cells were grown with vigorous shaking at 37°C and protein expression was induced with IPTG (1 mM). Induced cultures were incubated at 30°C until early stationary phase and cells were lysed using Bugbuster (Novagen). Recombinant proteins were purified using immobilized metal ion affinity chromatography on IMAC Sepharose™ 6 Fast Flow (GE Healthcare). The imino-diacetic acid ligand was charged with 0.5 M NiSO_4_·6H_2_O and equilibrated with 50 mM HEPES (pH 7.4) containing 0.5 M NaCl, 10% glycerol, 0.1% Triton X-100 and 5 mM imidazole. Recombinant protein fragments were eluted using 150 mM imidazole in the same buffer and proteins were resolved by SDS-PAGE with 8% acrylamide. Full-length SspB was purified from inclusion bodies that were prepared using Bugbuster (Novagen) according to the manufacturer's instructions. Inclusion bodies were dissolved in 8 M urea supplemented with 0.1% Sarkosyl, then diluted to 3.4 M urea in 50 mM HEPES buffer (pH 7.4) containing 0.1% Sarkosyl. Recombinant SspB was refolded *in situ* on the IMAC column by washing with 50 mM HEPES (pH 7.4) containing 0.1% Sarkosyl, followed by further washes with 50 mM HEPES (pH 7.4) containing 0.1% Triton X-100 and 5 mM imidazole. SspB was eluted in 50 mM HEPES (pH 7.4), 0.1% Triton X-100 and 150 mM imidazole.

### Metal ion analysis

Recombinant protein fragments of AspA and SspB (100 ng µl^−1^ in 50 mM HEPES, pH 7.4 containing 0.5 M NaCl, 10% glycerol and 0.1% Triton X-100) were analysed by ICP-OES (Perkin-Elmer Optima 3000). Calibration used the ICP multi-element standard solution IV (Merck) containing 23 elements (Ag, Al, B, Ba, Bi, Ca, Cd, Co, Cr, Cu, Fe, Ga, In, K, Li, Mg, Mn, Na, Ni, Pb, Sr, Tl, Zn) in dilute nitric acid. The protein preparation was assayed for the presence of Cu, Fe, Mn, Mg, Ni and Zn, the concentrations of which were calculated as molar ratios and normalized for the buffer alone.

### Cell surface protein extraction and Western blotting

Proteins were extracted from the surface of *S. pyogenes* cells by modification of the method utilized to extract surface proteins from *S. gordonii* and *L. lactis* (Jakubovics *et al*., 2005). The modification involved incubating cells suspended in spheroplasting buffer with mutanolysin (500 U) and lysozyme (3 mg ml^−1^) for 2 h at 37°C. Proteins were solubilized in SDS-containing buffer without 2-mercaptoethanol and separated by SDS-PAGE. Polypeptides were transferred onto nitrocellulose membrane by Western electroblotting. Blots were blocked with 1% bovine serum albumin (BSA) or 1% skimmed milk powder in Tris-buffered saline supplemented with 0.1% Tween 20 (TTBS) and then incubated with primary antibody at appropriate dilution. Antibody binding was detected with HRP-linked goat anti-rabbit IgG, and blots were developed using 4-chloro-1-naphthol or by ECL (Amersham). Antibodies to AspA-VP were raised in New Zealand White rabbits (Covalab) and anti-SpaP rabbit antibodies were kindly provided by Charles Kelly (Kings College London). Both antisera were routinely used at 1:1000 dilution.

### *In vitro* gp-340 binding assays

Gp-340 was prepared from parotid saliva samples pooled from multiple donors using a multi-step procedure including adsorption onto *S. mutans* as described previously (Loimaranta *et al*., 2005). Adherence of *L. lactis* cells expressing AspA or SspB to immobilized gp-340 was carried out by crystal violet assay and data converted to cell numbers as previously described (Jakubovics *et al*., 2005). Immulon 2HB 96-well microtitre plates were coated with 50 ng gp-340 in coating buffer (20 mM Na_2_CO_3_, 20 mM NaHCO_3_; pH 9.3) as this gp-340 concentration was found to be optimal for the assay. For antibody inhibition studies, *L. lactis* cells expressing AspA, or *L. lactis* MG1363 controls, were incubated with dilutions of various antisera for 30 min at 25°C before being assayed directly for binding to immobilized gp-340. Absorbance values were corrected against those obtained with corresponding *L. lactis* control cells taken through the entire procedure.

### Transformation of *L. lactis* MG1363

Plasmid pKS80 expressing SspB (pKS80 *sspB*^+^) was produced by PCR (Expand Long PCR, Roche) of *sspB* (GenBank Accession No. U40027) using primer pairs BamH1F & BamH1Rev and BamSspF2 & BamH1Rev (Table S2) to remove the internal BamHI site. The *sspB* fragment was subcloned into pKS80 using BamHI and SbfI. Plasmid pKS80 *aspA*^+^ was produced by PCR amplification of the 4.5 kb region corresponding to *aspA* from *S. pyogenes* H360 (which shares 100% identity with *spy1325*/*aspA* from MGAS6180) using primers AgI/II-pKSF and AgI/II-pKSR (Table S1), and subcloning into pKS80 using BamHI and PstI.

Electrocompetent cells of *L. lactis* were prepared by culturing in GM17G broth (M17 broth supplemented with 10% glycine and 0.5% glucose) at 30°C to OD_600_ = 0.5–0.6. Cultures were incubated on ice for 10 min and cells harvested by centrifugation (3000 *g*, 4°C, 10 min). Cells were suspended in electroporation buffer (0.5 M glucose, 10% glycerol) and electroporated using a Bio-Rad Genepulser II at 1 kV voltage, 50 µF capacitance and 100 Ω resistance. Transformants were recovered in modified M17 medium (M17 containing 0.5% glucose, 0.5 M sucrose, 20 mM MgCl_2_ and 2 mM CaCl_2_), plated onto M17 agar supplemented with appropriate antibiotics, and incubated for up to 48 h at 30°C in a candle jar. Expression of SspB or AspA on the surface of *L. lactis* was verified by SDS-PAGE and Western immunoblot analyses. Expression levels of SspB and AspA were similar as visualized on Coomassie blue-stained gels.

### Far-Western analysis of gp-340 binding to recombinant AspA protein fragments

Proteins were transferred from SDS-PAGE gels to PVDF membrane by electroblotting at 50 V for 2 h. Membranes were blocked for 16 h in Tris-buffered saline containing 0.1% Tween 20 and 1% BSA (TTBS-BSA). The membrane was incubated at 20°C for 1 h in TTBS-BSA containing 0.5 µg of purified gp-340 (Loimaranta *et al*., 2005) and washed twice with TTBS. Mouse monoclonal primary anti-gp-340 antibody (BioPorto Diagnostics, Denmark) in TTBS-BSA was incubated with the membrane for 1 h at 20°C and the membrane washed as before. Antibody binding was detected using HRP-conjugated goat polyclonal anti-mouse antibody (DakoCytomation) and blots were developed with 4-chloro-1-naphthol. The monoclonal primary anti-gp-340 antibody or secondary antibody did not react directly with the AgI/II protein bands.

### Gp-340- or peptide SRCRP2-mediated aggregation assays

*Lactococcus lactis* strains were grown in C medium and harvested by centrifugation at 5000 *g* for 10 min. The OD_600_ was adjusted to 0.6, and aggregation assays were carried out in triplicate as previously described (Jakubovics *et al*., 2005). Gp-340 or SRCRP2 (16-mer peptide; QGRVEVLYRGSWGTVC) in PBS were added to cell suspensions (final concentration 0.5 µg ml^−1^). These were incubated at 37°C and OD_600_ readings taken at regular intervals over a period of 5 h. Controls containing no gp-340 or SRCRP2 were analysed simultaneously. Aggregation was expressed as a percentage decrease in OD_600_ relative to 100% aggregation.

### Generation of *aspA* knockout mutants

Knockout mutants of strains H360 and MGAS6180 were generated through allelic replacement of *aspA* with *aad9* (encoding spectinomycin resistance), and were complemented *in trans* with pKS80 *aspA*^+^ plasmid. The mechanism for generating the mutants is shown in Fig. S2A, based upon the MGAS6180 sequence. A 1045 bp region directly upstream of *aspA* and a 1051 bp region directly downstream were amplified using primers US1325-F & US1325-R, DS1325-F & DS1325-R respectively, which introduced BamHI sites into the products (Table S2). These fragments were stitched together in a second round of PCR using primers US1325-F & DS1325-R (Table S2) that generated a 2 kb fragment with a central BamHI site. This PCR product was ligated into pGEM-T (Promega), generating pGEM-T::*aspA*-2kb, and this plasmid was transformed into *E. coli* XL1. The *aad9* gene, encoding spectinomycin resistance, was PCR-amplified using primers Aad9fwd & Aad9rev (Table S2) and then subcloned into pGEM-T::*aspA*-2kb at the BamHI site, thus interrupting the truncated *aspA* fragment and providing a means of positive selection of transformants (Fig. S2A). The resulting plasmid construct, designated pGEM-T::*aspA-aad9*, was transformed into GAS by electroporation as described above. Transformants were recovered on THY agar supplemented with spectinomycin at 37°C in 5% CO_2_ for up to 48 h. Correct insertions were identified by PCR of chromosomal DNA with primers US1325-F & DS1325-R, and the products were sequenced to confirm authenticity.

### Saliva collection

Whole saliva was collected from five to eight healthy volunteers. Following collection, saliva was pooled and dithiothreitol added to a final concentration of 2.5 mM. Particulate matter and mucins were removed by centrifugation at 13 000 *g* for 10 min. Clarified saliva was diluted to 10% concentration with distilled water, passed through a 0.2 µm nitrocellulose filter and stored at −20°C. Local guidelines for collection, storage and disposal were followed in accordance with the Human Tissue Act.

### Static biofilm models

Strains were initially grown in C medium for 16 h in 5% CO_2_, cells were harvested by centrifugation, and suspensions adjusted to OD_600_ = 0.1 in modified C medium (0.25% Difco Proteose Peptone #2, 0.75% yeast extract, 10 mM K_2_HPO_4_, 0.4 mM MgSO_4_, 17 mM NaCl, pH 7.5) containing 0.2% glucose. Biofilms were grown on sterile 13-mm-diameter glass coverslips (Menzel-Glaser, Germany) that had been incubated with 0.5 ml of 10% saliva (4°C for 16 h) in 24-well microtitre plates (Greiner). Cell suspensions (0.5 ml) were added to wells containing saliva-coated coverslips and incubated at 37°C under 5% CO_2_ for 24 h. To estimate biomass, biofilms on glass coverslips were washed twice with PBS and stained with crystal violet for 10 min. After washing with distilled water the biofilm-coated coverslips were transferred to a fresh 24-well plate, crystal violet was solubilized with 7% acetic acid, and absorbance at 595 nm (*A*_595_) measured as an indicator of biomass (Jakubovics *et al*., 2005).

In separate experiments to compare biofilm formation on different substrata, gp-340 (50 ng) or saliva (10%) was adsorbed onto the surface of Immulon 2HB 96-well microtitre plates. Non-specific binding sites were blocked with 3% BSA and the wells washed in TBS. *S. pyogenes* strains were grown in C medium for 16 h at 37°C in 5% CO_2_, harvested by centrifugation, and cell suspensions adjusted to OD_600_ = 1.0 in modified C medium containing 0.2% glucose. Suspensions (0.1 ml) were applied to triplicate wells and incubated for 3 h at 37°C in 5% CO_2_. Suspensions were then aspirated and the wells washed in TBS before fresh modified C medium/0.2% glucose (0.2 ml) was added to each well. Plates were incubated for a further 20 h, after which time wells were washed with TBS and the biofilms stained with 0.5% crystal violet for 2 min. Excess stain was removed by washing in TBS, remaining crystal violet solubilized in 10% acetic acid (0.1 ml), and *A*_595_ values were determined as before.

### Confocal microscopy

Mature 24 h biofilms, grown as described above, were washed with PBS and fixed with 0.5 ml of 4% paraformaldehyde for 1 h at room temperature. Biofilm-coated coverslips were washed with PBS prior to staining with fluorescein isothiocyanate (FITC; 1.5 mM final concentration in 0.05 M Na_2_CO_3_ containing 0.1 M NaCl) for 1 h with gentle agitation in the dark. Washed biofilm-coated coverslips were mounted onto microscope slides in 5 µl of Vectashield mounting medium containing DAPI (Vectorlabs) and sealed onto the slide with varnish. Biofilms were visualized using a Leica TCS-SP2 confocal imaging system attached to a Leica DMIRBE inverted microscope. Analysis of the data was carried out using Volocity image analysis software (Improvision).

### Statistical analysis

All data are reported as mean ± standard deviation (SD) unless otherwise indicated. Significance between samples was determined using the paired two-tailed Student's *t*-test, and a value of *P* < 0.05 was accepted as indicating significance. Data were analysed with GraphPad Prism v5 software.
