# Supplementary material for: Streptococcus pyogenes antigen I/II-family polypeptide AspA shows differential ligand-binding properties and mediates biofilm formation
Source: Mol Microbiol. 2011 Jul 15;81(4):1034–49. doi: 10.1111/j.1365-2958.2011.07749.x (PMC3178794; doi:10.1111/j.1365-2958.2011.07749.x)
Supplement: Supplementary file 1 [file mmi0081-1034-SD1.pdf]

```
                                   AspA-F1 →
AspA   1     MKQMETKGYGYFRKTKAYGLVCGIT--------------LAGALTLGTTSVSADDVTTLNPATNLTTLQTPPTADQTQLAHQAGQQSGELVSEVSNTEWD   86
SspB   1     MQKREV--FG-FRKSKVAKTLCGAV-LGAALIAIADQQVLADEVTETNSTANVAVTTTGNPATNLPEAQGEATEAASQSQAQAGSKDGALPVEVSADDLN   96

AspA   87    NAVTTAQKAGVTVKQSEKVTHDSLSSAQADLEKQTQAVTEATTKQEANTTAINQAVSENKAIDQANRDEKARVDALNTKGEADTKAKNETGQAQVDAQNK   186
SspB   97    KAVTDAKAAGVNVVQDQTSDKGTATTAAENAQKQAEIKSDYAKQAEEIKKTTEAYKKEVEAHQAETDKINAENKAAEDKYQEDLKAHQAEVEKINTAN-A   195

AspA   187   QAQAAADAMNAKLKADYEAKLTEIKQIESENEAIRQRNQQASQATHQANQAAQAAYQEKLAEIERIKAENAAIRDRNAKAQQEAERQNQALQAAYEAKLA   286
SspB   196   TAKAEYEAKLAQYQKDLAAVQKANEDSQLDYQNKLSAYQAELARVQKANAEAKEAYEKAVKENTAKNAALQAENEAIKQRNETAKANYDAAMKQYEADLA   295
                     SGP1                                SGP2                                        SGP1

AspA   287   EIKQIESEN--------AAIRQRNEQAGQATNQTNQAAQAAYQEKLAEIERIKAGNAAIRDRNAKAQQEAERQNQALQAAYEAKLAEIKQIESENAAIRK   378
SspB   296   AIKKAKEDNDADYQAKLAAYQAELARVQKANADAKAAYEKAVEENTAKNTAIQAENEAIKQRNETAKATYEAAVKQYEADLAAVKQANATNEADYQAKLA   395
                                    SGP2                               SGP1

AspA   379   RNEQAGQTTNQTNQAAQAAYQEKLAEIERINAENAAIRDRNTKARQEAERQNQQLKTTYEAQLSAYQQALQAK-----------------KEAENKAIDQ   461
SspB   396   AYQTELARVQKANADAKATYEKAVEDNKAKNAALQAENEEIKQRNAAAKTDYEAKLAKYEADLAKYKKDFAAY----------T----AALAEAESKKKQ   481
                    SGP2

                     AspA-mF →
AspA   462   VVFGIDAKANGVDNAEYGNSIMTVTTQPDGSFVFKHDMIDGVKTIGHGTLTGKIN------------------------------------------HHY   519
SspB   482   DGYLSEPRSQSLNFKSEPNAIRTIDSSVHQYGQQELDALVKSWGISPTNPDRK-------------KS-------------TAYSYFNAINSNNTYAKLV   555

AspA   520   EAN-------KDGSITAYIDSVTFDKYEYQNVAKNDAVDKNIAFRILSATGQELFVKAHDGDKTFS------------------------------ET-L   581
SspB   556   LEKDKPVDVTYTGLKNSSFNGKKISKVVYTYTLKETGFNDGTKMTMFASSDPTVTAWYNDYFTSTN-------INVKVKFYDEEGQLMNLTGGLVNFSSL   648

AspA   582   N----------------KTVSLKLTYQLKPHEAVKDIKVFQLHDDWVHDTHG------------------------------------------------   617
SspB   649   NRGNGSGAIDKDAIESVRNFNGRYIPISGSSIKIHENNSAYADSSNAEKSRG--------ARWDTSEWDTTSSPNNWYGA------------------VG   722

                                                   ← AspA R3
AspA   618   --------------SALVSYVNNNDAVPHIEVPE-KP--VEPDMVTPKVEQEKPVPEAPQKPTDGVPNLEKEKPVPPTPVKPEAVKPVLEQEKPVPEAPQ   700
SspB   723   EITQSEISFNMASSKSGNIWFAFNSNINAIGVP--T-----KPVAPTAPTQPMYETEKPLEPAPVVPTYENE-PTPPVKTPDQPEPSKPEEPTYETEKPL   814

AspA   701   KPTDDVPNLEKEKPVPPTPVKPEAVKPVLEQEKPVPEAPQKPTDDVPNLEKE-KPVPPTPVEPEAIKPDLQSFTPEVYDPIKPVVKPHVTVPEKVVYEVM   799
SspB   815   EPAPVAPTYENE-PTPPVKIPDQPEPSKPEEPTYETEKPLEPAPVAPTYENEPTPPVKTPDQPEPSKPEEPTYDPLPTPPLAPTPKQLPTP--PVVPTVH   911

                     AspA-F3 →
AspA   801   VHPVQVKQTPTNVKSVTNSDQVNIDGQLVPKGSTVTWELVNTSLKAGRQDITSYELTDPLPDGFELDVTATQTLSPEWVITTDEAG-KVSLKASQSLLAH   898
SspB   912   FHYSSLLAQPQINKEIKNEDGVDIDRTLVAKQSIVKFELKTEALTAGRPKTTSFVLVDPLPTGYKFDLDATKAASTGFDTTYDEASHTVTFKATDETLAT   1011
                                                ADH1

AspA   900   FNAKRDQDVEVPKVSLVGRLLNDAGTYHNTFKTVVTTPTGSYTVISNTPVIYTPGNDPKTPRNPGGDNPTPHDNLIQPTKTIVDDKGQSIDGKSVLPNST   1005
SspB   1012  YNADLTKPVETLHPTVVGRVLNDGATYTNNFTLTVNDAYGIKSNVVRVTTPGKP-NDPDNPNN----------NYIKPTKVNKNKEGLNIDGKEVLAGST   1101
                     ADH2
```

```
                           ←—— AspA-mR
AspA   1006   LTYVAKQDFDQYKGMTAAKESVMKGFIYVDDYKDEAIDGHSLVVNSIKAANGDDVTNLLEMRHVLSQDTLDDKLKALIKASGISPVGEFYMWVAKDPAAF   1105
SspB   1102   NYYELTWDLDQYKGDKSSKEAIQNGFYYVDDYPEEALDVRPDLVKVADEKG--NQVSGVSVQQYDSLEAAPKKVQDLLKKANITVKGAFQLFSADNPEEF   1199
                                                                                        BAR

AspA   1106   YKAYVQKGLDITYNLSFKLKQDFKKG--DITNQTYQIDFGNGYYGNIVVNHLSELTVHKDVFDKEG--GQSINAGTVKVGDEVTYRLEGWVVPTNRGYDL   1201
SspB   1200   YKQYVSTGTSLVITDPMTVKSEFGKTGGKYENKAYQIDFGNGYATEVVVNNVPKITPKKDVTVSLDPTSENLDGQTVQLYQTFNYRLIGGLIPQNHSEEL   1299

AspA   1202   TEYKFVDQLQHTHDLYQK-DKVLATVDITLSDGSVITKGTDLAKYTETVYNKETGHYELAFKQDFLAKVVRSSEFGADAFVVVKRIKAGDVANEYTLYVN   1300
SspB   1300   EDYSFVDDYDQAGDQYTGNYKTFSSLNLTMKDGSVIKAGTDLTSQTTAETDATNGIVTVRFKEDFLQKISLDSPFQAETYLQMRRIAIGTFENTYVNTVN   1400

                                                                                  ←—— AspA-R1
AspA   1301   GNPVKSNKVTTHTPE-Q------------P----QPVTPKAP----------------------ALPSTGEQGVSILTALGAALLSLLGYVGLKKRQQ--   1352
SspB   1401   KVAYASNTVRTTTPIPRTPDKPTPIPTPKPKDPDKPETPKEPKVPSPKVEDPSAPIPVSVGKELTTLPKTGTNDATYMPYLGLAALVGFLGLGLAKRKED-   1500
```

Supplemental Fig. S1. Alignment of *S. pyogenes* AspA amino acid sequence with *S. gordonii* SspB. Positions of primers (Table S2) are shown above the sequences.

Key to underlines: SGP1 and SGP2, salivary glycoprotein-binding regions in SspB; ADH1 and ADH2, salivary glycoprotein-adherence mediating regions in SspB; BAR, sequence in SspB recognized by *Porphyromonas gingivalis*

Key to amino acids: Red text with yellow background, identical amino acids; black text with green background, similar amino acids; green text with white background, weakly similar amino acids; black text with white background, non-identical/non-similar amino acids.

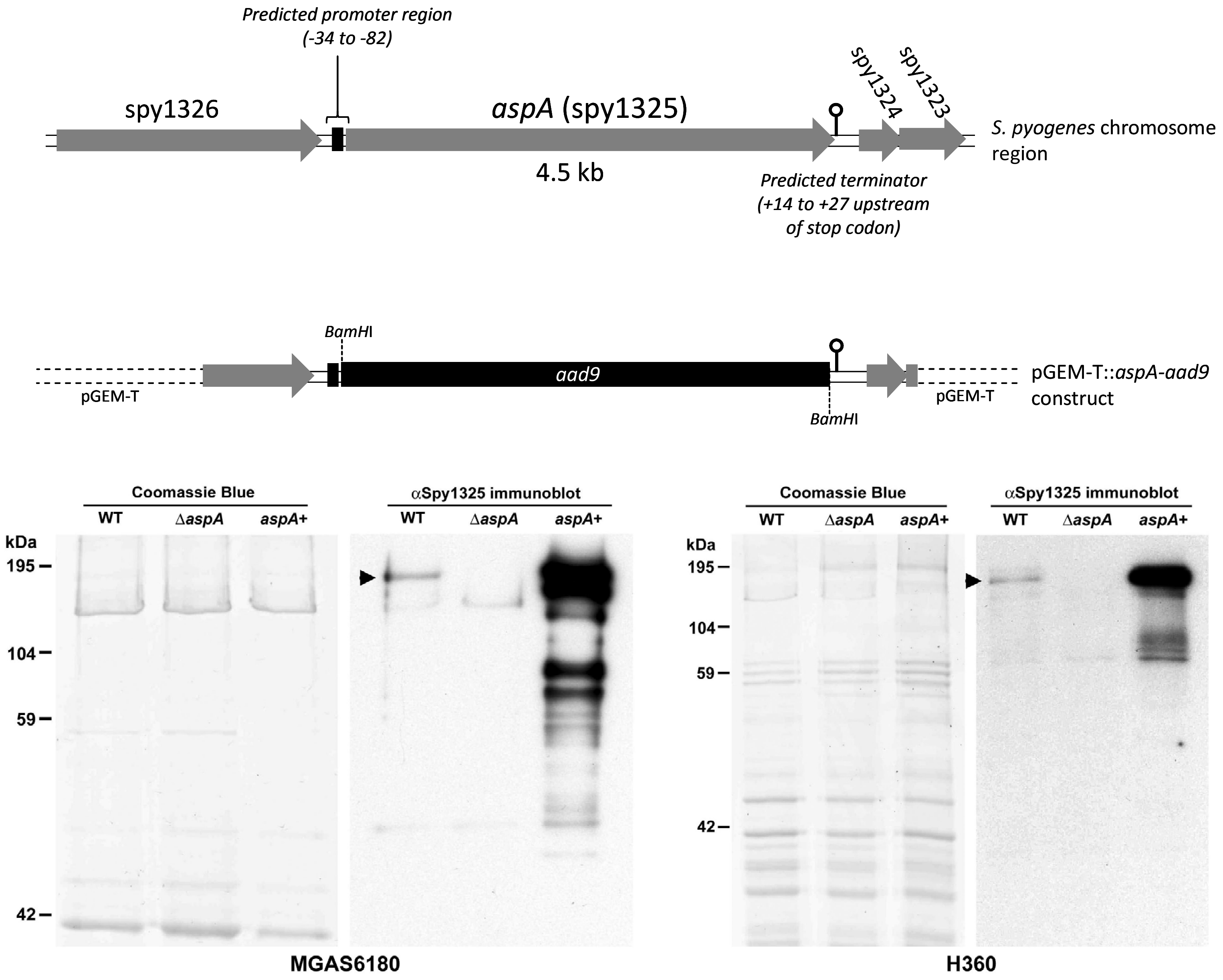
Predicted promoter region
(-34 to -82)
spy1326
aspA (spy1325)
4.5 kb
spy1324
spy1323
S. pyogenes chromosome region
Predicted terminator
(+14 to +27 upstream of stop codon)
BamHI
aad9
pGEM-T
pGEM-T
BamHI
pGEM-T::aspA-aad9 construct
Coomassie Blue
WT
ΔaspA
aspA+
αSpy1325 immunoblot
WT
ΔaspA
aspA+
kDa
195
104
59
42
MGAS6180
Coomassie Blue
WT
ΔaspA
aspA+
αSpy1325 immunoblot
WT
ΔaspA
aspA+
kDa
195
104
59
42
H360

Supplemental Fig. S2. Production of isogenic *aspA* knockout mutants in strains MGAS6180 and H360, and AspA expression profiles of wild-type, Δ*aspA* and complemented strains.

(A) Regions flanking the *aspA* gene, which were identical in the two strains, were PCR-amplified and the *aad9* cassette conferring spectinomycin resistance was ligated in between the flanking regions. The construct was transformed into GAS, thus deleting the *aspA* gene by allelic replacement. The knockout mutants were complemented *in trans* with pKS80 *aspA*$^+$ plasmid. The *aspA* gene (4.5 kb) lies downstream of M28_*spy1236* and upstream of two small operonic genes, M28_*spy1234* and M28_*spy1323*. The *aspA* gene is mono-cistronic with its own promoter region and a rho-independent terminator sequence. The putative *aspA* promoter region is located between 34-82 bp upstream of the ATG start codon, and putative terminator lies between 14-27 bp downstream of the stop codon. The allelic replacement resulted in deletion of the complete coding sequence, leaving in place the native promoter and terminator, as confirmed by sequencing.

(B) Western immunoblot analysis of cell wall-extracted proteins from *S. pyogenes* MGAS6180 or H360 wild-type strains, Δ*aspA* mutants, and Δ*aspA* (pKS80 *aspA*+) complemented strains. Blots were reacted with rVP-AspA antiserum (1:500 dilution) and antibody binding was detected with HRP-linked secondary antibody followed by ECL. Corresponding Coomassie Blue stained gels are shown and an equivalent amount of protein (5 µg) was applied to each lane.

Table S1. Bacterial strains used in this study

**Strain**		**Characteristics**	**Reference/Source**
E. coli	JM109	*recA*	Novagen
	BL21/λDE3	Lamba DE3 lysogen	Novagen
	XL1	*lacI*^{q} Δ*lacZ*	Stratagene
S. pyogenes	H360	Serotype M28, STSS*	Proft *et al.* (2003)[†]
	UB2042	H360 Δ*aspA::aad9*	This study
	UB2050	H360 Δ*aspA::aad9* (pKS80 *aspA*$^+$)	This study
	MGAS6180	Serotype M28, invasive disease	Green *et al.* (2006) [‡]
	UB2086	MGAS6180 Δ*aspA::aad9*	This study
	UB2117	MGAS6180 Δ*aspA::aad9* (pKS80 *aspA*$^+$)	This study
L. lactis	MG1363	Wild-type	Laboratory stock
	UB2265	(pKS80 *aspA*$^+$)	This study
	UB2136	(pKS80 *sspB*$^+$)	This study

* Streptococcal toxic shock syndrome

[†] Proft, T., Sriskandan, S., Yang, L., and Fraser, J. D. (2003) Superantigens and streptococcal toxic shock syndrome. *Emerg Infect Dis* **9:** 1211-1218.

[‡] Green, N.M., Zhang, S., Porcella, S.F., Nagiec, M.J., Barbian, K.D., Beres, S.B., *et al.* (2005) Genome sequence of a serotype M28 strain of Group A *Streptococcus*: potential new insights into puerperal sepsis and bacterial disease specificity. *J Infect Dis* **192:** 760-770.

Table S2. Primers used in this study

Primer name	Primer sequence
AspA-F1	**GACGACGACAAGAT**GTTGGGTACAACAAGT
AspA-R3	**GAGGAGAAGCCCGGT**TATTTTTCTTGCTCAAC
AspA-mF	**GACGACGACAAGAT**GACTGTTACAAC
AspA-mR	**GAGGAGAAGCCCGGT**TACTTAGCCACGT
AspA-F3	**GACGACGACAAGAT**GGATCAGGTTAATATC
AspA-R1	**GAGGAGAAGCCCGGT**TACTCTCCTGTGGATGG
BamHI-F	AAAACCAAAAGACCCTGACAAACC
KSspBrev2	GCACTCGCCTGCAGGGTAATCAGTCCAAGCTATTTGATT
BamSspF2	GCACGCGGATCCAAATGGAAAAAAAAGATTATCTCA
BamHIRev	GGTTTGTCAGGGTCTTTTGGTTTT
AgI/II-pKSF	AAAGGAGGATCCAAATGAAACAAATGGAAACTAAGGGTTA
AgI/II-pKSR	TCGACTGCAGTCCATTAATAACTTACTGTTGACG
Aad9fwd	ATGCGGATCCAGTATAATAACTATAACTAATAACG
Aad9rev	CGTAGGATCCCTTTACCAATTAGAATGAATATTTCCCAAA
US1325-F	GCCAGCAGTCAGGGGAGTTGG
US1325-R	CTGAGGATCCCATGCGATCACGAATGGCGGCATTC
DS1325-F	GATGGGATCCTCAGAACCATTGTGGACGACAAGGG
DS1325-R	ATACTCCTTGCTCTCCTGTGG

Primer sequences in bold correspond to those required for cloning into pET46-Ek-LIC (Novagen). Underlined primer sequences correspond to restriction endonuclease sites.
